# Feature Reduction in Graph Analysis

**DOI:** 10.3390/s8084758

**Published:** 2008-08-19

**Authors:** Rapepun Piriyakul, Punpiti Piamsa-nga

**Affiliations:** Department of Computer Engineering, Faculty of Engineering, Kasetsart University, Jatujak, Bangkok, 10900, Thailand; Tel.: +66-29428555 ext 1419; Fax: +66-25796245; E-mail: rapepunnight@yahoo.com

**Keywords:** Path Analysis, Graph Analysis, Feature Selection, Mammogram

## Abstract

A common approach to improve medical image classification is to add more features to the classifiers; however, this increases the time required for preprocessing raw data and training the classifiers, and the increase in features is not always beneficial. The number of commonly used features in the literature for training of image feature classifiers is over 50. Existing algorithms for selecting a subset of available features for image analysis fail to adequately eliminate redundant features. This paper presents a new selection algorithm based on graph analysis of interactions among features and between features to classifier decision. A modification of path analysis is done by applying regression analysis, multiple logistic and posterior Bayesian inference in order to eliminate features that provide the same contributions. A database of 113 mammograms from the Mammographic Image Analysis Society was used in the experiments. Tested on two classifiers – ANN and logistic regression – cancer detection accuracy (true positive and false-positive rates) using a 13-feature set selected by our algorithm yielded substantially similar accuracy as using a 26-feature set selected by SFS and results using all 50-features. However, the 13-feature greatly reduced the amount of computation needed.

## 1. Introduction

Breast cancer is among the most frequent forms of cancers found in women [9]. Diagnosis of breast cancer typically includes biopsy, ultrasound, and/or imaging. Ultrasound can diagnose simple cysts in the breast with an accuracy of 96-100% [11]; however, the unequivocal differentiation between solid benign and malignant masses by ultrasound has proven to be difficult. Despite considerable efforts toward improving ultrasound, better imaging techniques are still necessary. Mammography is now commonly used in combination with computer-aided diagnosis (CAD). CAD is a computer diagnosis system to assist the radiologists in image interpretation [15] Since the causes of some types of cancer are still unknown, it can be difficult to decide whether a tissue is cancerous or not. Currently, radiologists can refer to an automated system as a second opinion to help distinguish malignant from normal healthy tissues. An automated system can detect and diagnose probable malignancy in suspicious regions of medical images for further evaluation. Since medical images for CAD (such as X-ray, CT scan, MRI, and mammogram), include a considerable number of image features, CAD improves the detection of suspected malignancies.

Image features are conceptual descriptions of images that are needed in image processing for analyzing image content or meaning. Features are usually represented as data structures of directly extractable information, such as colors, grays, and higher derivatives from mathematical computation of the basic features such as its edges, histograms, and Fourier descriptors. Each type of feature requires a specific algorithm to process it. Therefore, only features that carry essential and non- redundant information about an image should be considered. Moreover, feature-extraction techniques should be practical and feasible to compute. Many researchers have tried to improve the accuracy of CAD by introducing more features on the assumption that this will lead to better precision. However, adding more features necessarily increases the cost and computation time.

The addition of more features does not always improve system efficiency, which has led to an investigation of feature pruning techniques [2, 3, 6, 20, 23, 30]. Foggia *et al.* [20] used a graph based method with only six features and found the performance was 82.83% true positive (TP) and 0.08% false positive (FP) per image, Fu *et al.* [13] used sequential forward search (SFS) and found that only 25 features are required, with Mean Square Error (MSE) 0.02994 by using General Regression Neural Networks (GRNN). When a support vector machine (SVM) was applied, it further reduced this to 11 features, with MSE of 0.0283.

Among the algorithms to discard non-significant features are sequential forward search (SFS), sequential backward search (SBF), and stepwise regression. SFS and SBF focus on the reduction of MSE of the detection process while stepwise regression involves both the interaction of features and the MSE value. Using stepwise logistic regression is costly since this technique is based on calculations over all possible permutations of every feature in the prediction model. These techniques use an assumption to select features that has higher relation to the classifier decision output. However, an optimal set of features must be orthogonal. With the above techniques, it is possible that information from two or more candidate features may be redundant and a feature may be dependent on another.

To improve the effectiveness of feature-discarding techniques, we propose a new method using modified path analysis for feature pruning. A weighted dependency graph of features to the output of classifier and correlation matrices among features is constructed. Statistical quantitative analysis methods (regressions and posterior Bayes) and hypothesis testing are used to determine the effectiveness of each feature in the classifier decision. Experiments are performed using 50 features found in literature and evaluate feature selection effectiveness when applied on to two learning models: ANN and logistic regression. The resulting 13-feature set is compared with prediction using all 50 original features and a 26-feature set selected by the SFS method. We found that the quality is nearly equal; however, the number of feature computations is reduced by one-half and 13/50 when compared to the 26-feature set and all-feature set, respectively.

The paper is organized as follows. Section 2 is the medical image features problems and survey on the features in medical image research. Section 3 describes the feature extraction domains. Section 4 has details of the statistical collaborative methods. Section 5 describes our proposed algorithm and section 6 is the evaluation the experiments.

## 2. Medical Image Feature Survey

Medical image detection from mammograms is limited to analysis of gray-scale features. Distinction between normal and malignant tissue by image density is nearly impossible because of the minuteness of the differences [20]. Thus, most feature extraction methods are extended from the derivation of limited gray scale information [1, 2, 10, 27, 30]. Medical image features can be divided into three domains: spatial, texture, and spectral. Spatial domain refers to the gray-level information in an arbitrary window size. It includes gray levels, background and foreground information, shape features, and other statistics derived from image information intensity. Texture refers to properties that represent the surface or structure of an object in reflective and transmissive images. Texture analysis is important in many applications of computer image analysis for classification, detection or segmentation of images based on local spatial variations of intensity. Spectral density or spectrum of signal is a positive real value function of a frequency associated with a stationary stochastic process, which has dimensions of power or energy. However, all useful features must be represented in a computable form.

In a previous study [12], we found that most features were extracted on the assumption that more features would enhance the detection system. There are many ways to extract new features such as modifying old features, using more knowledge from syntactic images [19], and using a knowledge base [18]. Much research has been devoted to finding the best feature or best combination of features that gives highest classification rate using appropriate classifier. Some perspectives on the situation of feature extraction and selection are reviewed next.

Fu *et al.* [13] used 61 features to select a best subset of features that produced optimal identification of microcalcification using sequential forward search (SFS) and sequential backward search (SBS) reduction followed by a General Regression Neural Network (GRNN) and Support Vector Machine (SVM). W found inconsistency between the results of the two methods *i.e.* a feature which was in the top-five most significant using the SFS but was discarded by the SBS.

Zhang *et al.* [21] attempted to develop feature selection based on the neural-genetic algorithm. Each individual in the population represents a candidate solution to the feature subset selection problem. With 14 features on their experiment, there are 2^14^ possible feature subsets. The results showed that a few feature subsets (5 features) achieved the highest classification rate of 85%. In the case of a huge number of features and mammography, however, it is very costly to select features using the neural- genetic approach.

The Information Retrieval in Medical Applications (IRMA) [3] project used global, local, and structure features in their studies of lung cancer. The global features consist of anatomy of the object; a local feature is based on local pixel segment; and structural features operate on medical apriori knowledge on a higher level of semantics. In addition to the constraints of the global feature construction and lack of prior medical semantic knowledge, this procedure was quite difficult and costly.

The researchers' choices of medical image features depend on the objectives of the individual research. Cosit *et al.* [2], Chiou and Hwang [6], and Zoran [30] used simple statistical features on gray scale intensity, while Samuel *et al.* [5] used volume, sphericity, mean of gray level, standard deviation of gray level, gray level threshold, radius of mass sphere, maximum eccentricity, maximum circularity, and maximum compactness in their CAD system. Hening [18] used average gray scale, standard deviation, skewness, kurtosis, maximum and minimum of gray scale, and gray level histogram to identify and detect lung cancer. Shiraishi [12] studied 150 images from the Japanese Society of Radiological Technology (JSRT) database by using patient age, RMS of power spectrum, background image, degree of irregularity, full width at half maximum for inside of segment region. Lori *et al.* [4] studied on personal profile, region of interest properties, nodule size, and shape. Ping *et al.* [21] extended the new modified features, number of pixel in ROI, average gray level, energy, modified energy, entropy, modified entropy, standard deviation, modified standard deviation, skewness, modified skewness, contrast, average boundary gray level. A further investigation on using more features unrelated to medical image analysis, Windodo [23] explored fault diagnosis of induction motors to improve the feature extraction process by proposing a kernel trick. On his study, 76 features were calculated from 10 statistics in the time domain. These statistics are mean, RMS, shape factor, skewness, kurtosis, crest factor, entropy error, entropy estimation, histogram lower and histogram upper. We cannot discern their common methods of selecting features; however, we can conclude that they added more features in order to increase the efficiency of their methods. Table 1 shows a summary of the features and classifiers from previous studies.

Explorations of feature extraction analysis have been found that the effects of significant features can be direct or indirect and some features do not relate to the detection results at all. Therefore, ineffective and redundant features must be discarded.

## 3. Feature Domains

This section presents details on feature domains that are used for medical image classification. Generally, the original digital medical image is in the form of a gray-scale or multiple spectrum bitmap, consisting of integer values corresponding to properties *(i.e.* brightness, color) of the corresponding pixel of the sampling grid. Image information in the bitmap is accessible through the coordinates of a pixel with row and column indices. All features that can be extracted directly using mathematical or statistical models are categorized as low-level features. High-level features are summarized from low-level features, usually by machine-learning models. Much research in medical image analysis has to deal with low-level features in order to identify high-level features. In this research, we investigate several types of low-level features in order to identify mammograms as benign or malignant. The low-level features are separated into spatial, textural, and spectral domains.

The spatial domain is composed of features extracted and summarized directly from grid information. It implicitly contains spatial relations among semantically important parts of the image. Examples of spatial features are shapes, edges, foreground information, background information, contrasts and set of intensity statistics, such as mean, median, standard deviation, coefficient of variation, variance, skewness, kurtosis, entropy, and modified moment. In this research, we also use radian of mass.

Texture features are relations among pixels in a bitmap. Representation of texture features commonly uses co-occurrence matrices to describe their properties. The co-occurrence matrix of texture describes the repeated occurrence of gray-level configuration in an image. For a texture image, *P_φ,d_*(*a*, *b*), denotes the frequency that two pixels with gray levels *a*, *b* appear in the window separated by a distance *d* in direction *φ*.

The frequencies of co-occurrence as functions of angle and distance can be defined as:

$$\begin{array}{l} P_{0^{{}^{\circ}},\;d}(a,b)=\left|\;\left\{\;\left[\left(k,l),(m,n\right)\right]\in \text{D}:k-m=0,\left|l-n\right|=d,\;f(k,l)=a,\;f(m,n)=b\right\}\right|\\ P_{45^{{}^{\circ}},}\;d(a,b)=\left|\;\{\;[\left(k,l),(m,n\right)]\in \text{D}:(k-m=d),l-n=-d\vee (k-m=-d,l-n=d),f(k,l)=a,\;f(m,n)=b\}\right|\\ P_{90^{{}^{\circ}},d}(a,b)=\left|\;\{\;[\left(k,l),(m,n\right)]\in \text{D}:\left|k-m\right|=d,l-n=0,\;f(k,l)=a,\;f(m,n)=b\}\right|\\ P_{135^{{}^{\circ}},d}(a,b)=\left|\;\{\;[\left(k,l),(m,n\right)]\in \text{D}:(k-m=d,l-n=d)\vee (k-m=-d,l-n=-d),f(k,l)=a,f(m,n)=b\}\right| \end{array}$$

where | {…} | refers to set cardinality, *f*(·,·) is a gray value and ***D*** = (*M* × *N*) × (*M* × *N*)

In this paper, we take *φ* to be 0°, 45°, 90°, and 135°, and *d*=1. Examples of features in texture domain are:

Energy or angular second moment (an image homogeneity measure): $\sum_{a,b}{P^{2}}_{\phi ,d}(a,b)$
Entropy: $\sum_{a,b}P_{\phi ,d}(a,b)\log_{2}P_{\phi ,d}(a,b)$
Maximum probability: $\max_{a,b}\{P_{\phi ,d}(a,b)\}$
Contrast: ${\sum_{a,b}\left|a-b\right|}^{k}P_{\phi ,d}(a,b)$
Inverse difference moment: $\sum_{a,b,a\neq b}\frac{{P^{\text{λ}}}_{\phi ,d}}{{\left|a-b\right|}^{k}}$
Correlation (a measure of image linearity, linear direction structures in direction *ϕ* $\sum_{a,b,a\neq b}\frac{\left[\left(\text{ab})P_{\phi ,d}(a,b\right)\right]-{\text{μ}}_{x},{\text{μ}}_{y}}{{\text{σ}}_{x}{\text{σ}}_{y}}$ where *μ_x_, μ_y_, σ_x_, σ_y_* are means and standard deviations.

Spectral features [3] are used to describe the frequency characteristics of the input image. The features are based on transformation from the spatial and time domains. Most frequently-used spectral features are based on discrete cosine transform (DCT) and wavelets. Examples of features based on the frequency domain are:

Spectral entropy: $-\sum_{i}\sum_{j}\bar{X}(i,j)h(\bar{X}(i,j))$
Block activity: $A=-\sum_{i}\sum_{j}\left|\bar{X}(i,j)\right|$ where *i, j* are window size and $\bar{X}(i,j)=\frac{\left|\bar{X}(i,j)\right|}{A}$

The above features are frequently found in the literature of medical image analysis; there are many more features available.

## 4. Methodology

We hypothesize that using only one statistical method for classification will not be successful because of the restriction on measurement values of features and output. As this restriction, we investigate statistical techniques to fulfill the feature selection process. These statistical techniques consist of four parts: 1) feature classification, 2) path analysis, 3) exploration on relations among features and outputs, and 4) hypothesis testing. In the feature classification, we use correlation analysis to transform a number of features into a number of groups. In path analysis, the conceptual relations among different feature classes are constructed. Then, relations among features and between features and outputs are determined by three methods: logistic regression, simple regression, and multiple regression. Finally, hypotheses of feature relationships are tested by a Bayesian technique.

## 4.1. Feature classification

Since most low-level features are extracted from spatial and texture based, which are highlycorrelated, the feature selection strategy is subject to this limitation. The correlation coefficient is usedto analyze these features. The correlation coefficient *p* between random variables *x* and *y* is defined as $p(x,y)=\frac{\mathrm{cov}(x,y)}{\sqrt{V(x)V(y)}}$ where cov(*x*, *y*) denotes the covariance of *x* and *y*, *V*(*x*) and *V*(*y*) are variances of *x* and *y*. *p* is between -1 and 1, and *p* = 0 indicates no linear relation between *x* and *y*.

Correlation coefficients of features can be used to classify many highly related features into groups.

## 4.2. Path analysis

By the previous phase, we can identify groups of highly-related features. We find that the relationships of features within each group and relationships among groups to final output can be determined by path analysis.

Path analysis utilizes multiple regression analysis. Regression analysis is an analysis of causal models when single indicators are endogenous variables of the model. In a path model, there are two types of variables: exogenous and endogenous. Exogenous variables may be correlated and may have direct effects as well as indirect effects on endogenous variables. Causality is a relationship between an exogenous variable and endogenous variable(s); philosophical causation refers to the set of all particular “causal” relations.

**Figure 1.**
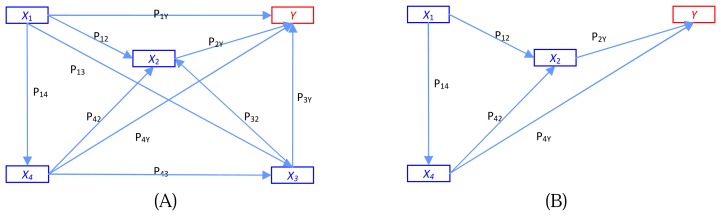
An example of a general recursive causal system with four independent features and a dependent output. (A) Illustration of possible relations among features and output. (B) The result of feature selection by analogy with graph base.

Being a regression-based technique, path analysis is limited by the requirement that all variables be continuous. Because our study involves continuous cause variables while the endogenous output variable is dichotomous (discrete), we cannot use path analysis directly; however, the analysis is still a graph-based process. Causal relation analysis can be explained by dependent variables that are measured on an interval or ratio scale [17]. Thus, for path analysis involving continuous endogenous variables, the categorical endogenous might have difficulty both in theoretical terms and prediction implication. Goodman [9] considered path analysis of binary variables by using logistic regression. Hagenaars [10] made a general discussion of path analysis of recursive causal systems of categorical variables by using the directed log-linear model approach, which is a combination of Goodman's approach and graphical modeling. Example of the different models of trait effects on output y is illustrated in Figure 1. Figure 1A shows a multiple regression model where each trait operates simultaneously on fitness y. Figure 1B is the path analysis model showing four traits at four time periods.

A path diagram not only shows the nature and direction of causal relationships but also estimates the strength of relationships. Comparatively weak relationships can be discarded; thus some features are eliminated. A path coefficient is the standardized slope of the regression model. This standardized coefficient is a Pearson product – moment correlation. Basically, these relationships are assumed to be unidirectional and linear. To overcome this limitation, we use regressions and Bayesian inference to construct a graphical model.

## 4.3. Relations among features and outputs

From the previous details about features and the path analysis, it is necessary to explore the cause and effect features by regression analysis. In our purpose, we suggest to use logistic regression, simple regression, and multiple regressions.

a) a) Using logistic regression. Logistic regression is a regression model for Bernoulli-distributed dependent variables. It is a linear model that utilizes the logit as its link function. Logistic regression has been used extensively in medical and social sciences [4, 11]. The logit model takes the form: $\log(\frac{p_{i}}{1-p_{i}})=\text{α}+{\text{β}}_{1}x_{1i}+{\text{β}}_{2}x_{2i}+\ldots +{\text{β}}_{k}x_{\text{ki}}+e_{i};i=1,2\ldots n,$
  where *p_i_*=Pr(y_i_=1), *β_j_*>0; *j* =1, 2 … *k* are parameters (weight) of feature *x_i_* and *e_i_* is a random error (bias) of feature vector of a sample data.
  Logistic regression model can be used to predict the response features to be 0 or 1 (benign or malignant in the case of mammogram detection). Rather than classifying an observation into one group or the other, logistic regression predicts the probability *p* of being in either group. The model predicts the log odds (*p*/(1-*p*)) that an observation later be transformed to *p* as value of 0 or 1 with an optimal threshold. The general prediction model is log(*p*/(1*-p*)) = **xβ***+***ϵ**, where **x** is feature vector; **β** is a parameter vector; and **ϵ** is a random error vector.
b) Using simple regression and multiple regression. Simple regression has the same basic concepts and assumptions as logistic regression but the dependent variable is continuous and the model has only a single independent variable. The simple regression can be modeled as *Y_i_* = *β*_0_ +*β*_1_*X*_1_*_i_* + *e_i_* ;*i* =1,2…*n* where *Y_i_* is the dependent variable, *β*_0_ , Regression yields a p value for the estimator of*β*_1_ are parameters (weights), and *n* is the size of training data. *X*_1_*_i_* is an explained variable of data record *i* and *e_i_* is a random error. Regression yields a *p* value for the estimator of Perform simple logistic regression *β*_1_ that can be used to decide whether *Y* has a linear relation to *X* . Multiple regression is an extension of simple regression model to multiple variables.

Simple logistic regression and multiple logistic regression are used to explore the cause features to effect output.

## 4.4. Hypothesis testing

Although the statistical techniques in previous Section can be used to identify causal features, they cannot classify those features as direct or indirect. We use hypothesis testing for this.

An appropriate way to test the hypothesis about the direction of causal relationships is easier to illustrate an abstract concept by analogy with Bayesian inference. Bayesian inference uses the scientific method, which involves collecting evidence that may or may not be relevant to a given phenomenon. The more evidence is accumulated, the degree of belief in a hypothesis changes. With enough evidence, the degree of belief will often become very high or very low. It can be used to discriminate conflicting hypotheses. Bayesian inference usually relies on degrees of belief, or subjective probabilities. Bayes's theorem adjusts probabilities based on new evidence as $P(H_{0}|E)=\frac{P(E|H_{0})P(H_{0})}{P(E)}$, where *H_o_* represents the hypothesis; *P*(*H_o_*) is the prior probability of *H_o_*; *P*(*E*\*H_o_*) is the conditional probability of availability the evidence *E* given that the hypothesis *H_o_* is true; and *P*(*E*) is the marginal probability of *E*, which is the probability of witnessing the new evidence *E* under all mutually exclusive hypotheses*. P*(*E*\*H_o_*) is the posterior probability of *H_o_* given *E*.

Using hypothesis testing on the regression, we can use path analysis for the discrete output.

## 5. Proposed Algorithm

To solve this solution, simple regression, logistic regression, and Bayesian inference take into account of causality extraction problem. The algorithm is described as following steps.

**Figure 2.**
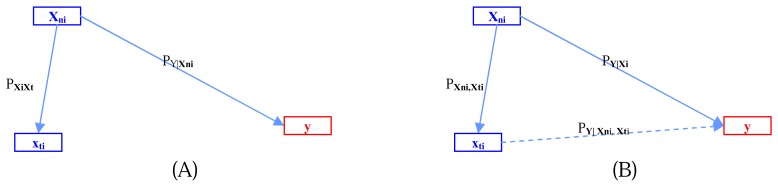
The connected graph on two cause features and effect y. There is no direct effect of feature x_ti_ on y in (A) but, as shows in (B), there is an interaction effect of feature x_ti_ in addition with x_ni_ on y.

Step 1: Partition the original feature sets (**x_1_**, **x_2_** … **x_n_**) into subsets using coefficients of the correlation matrix. Let the feature subsets be *S_i_* = (**x_1i_**, **x_2i_** … **x_ji_**), *i*=1, 2 … *k* with *p_ij_* being the correlation coefficient between **x_i_** and **x_j_**.
  This step is to partition all features into feature subsets *S_i_*, where *S_i_* and *S_j_* (*i≠j*) are lowly dependent based on the correlations.
Step 2: Perform simple logistic regression of each independent feature **x_ji_** ϵ *S_i_*, *j*=1, 2 … *R_i_* and dependent output **y** and then select the possible solution which satisfies a threshold value *P*.
  The result from this step is a subset *Ai* = (x_ri_, x_pi_ … x_ki_) of features from *S_i_* is where each element of *A_i_* is a direct causal feature of output y.
Step 3: Perform multiple logistic regression by using all features in set S_i_, i=1, 2 … k in the model and selecting the signified features B_i_ = (**x_ti_**, **x_li_** … **x_zi_**) from the model, where B_i_ is a set of direct features and indirect cause features.
Step 4: Let D_i_ = A_i_ Ə B_i_; where Ə is our testing hypothesis operator for exploring the causal relations using the Bayesian inference conceptual framework.
  This step is performed using Bayesian inference as in the following example for two features:
  (1) $$\begin{array}{ll} \text{If feature}\;{\text{x}}_{\text{ni}}\;\text{is the cause of}\;\text{y} & \approx P(\text{y}|{\text{x}}_{\text{ni}})\;>C \end{array}$$
  (2) $$\begin{array}{cc} \text{If feature}\;{\text{x}}_{\text{ti}}\;\text{is related}\;(\text{highly correlated})\;\text{to}\;{\text{x}}_{\text{ni}}\; & \approx P({\text{x}}_{\text{ni}}\;,{\text{x}}_{\text{ti}})\;>C \end{array}$$
  (3) $$\begin{array}{cc} \text{If feature}\;{\text{x}}_{\text{ti}}\;\text{is not significant to}\;\text{y} & \approx P(\text{y}|{\text{x}}_{\text{ti}}\;)<C \end{array}$$
  (4) $$\begin{array}{cc} \text{If feature}\;{\text{x}}_{\text{ni}}\;\text{and},{\text{x}}_{\text{ti}}\;\text{are significant to}\;\text{y} & \approx P(\text{y}|{\text{x}}_{\text{ni}}\;,x_{\text{ti}}\;)>C \end{array}$$
  where C is a given threshold
  This step iteratively refines the search for the indirect cause feature with the highest correlation with the direct cause **x_mi_**.
  Through the above predicates (1) to (4), we can accept the *hypothesis* that **x_ni_** and the combination of **x_ni_** and **x_ti_** cause **y**. Figure 2 illustrates the relations among **x_ni_**, **x_ti_**, and **y.**
Step 5: Repeat from Step 2 while *i* ≤ *k*. This step produces sets *D_i_,* where *i*=1, 2 … *k*. Note that some of *D_i_* may be null sets.
Step 6: Construct graph G by merging subgraphs *D_i_*; *i*=1, 2 … *k*;
  *G*(*V*, *E* | *Y*) = ∪*^k^_i_*_=1_*D_i_*;*V* = (*v_i_*); *E* = (*e_i_*); *Y* is the effect or dependent vertex.

## 6. Experiment and Results

### 6.1. Experiment

Our experiment is based on a training set of 113 ROIs from the Mammographic Image Analysis Society (MIAS) mammogram images that are segmented by radiologists. After image segmentation, 50 features from the spatial, texture, and spectral domains are extracted. The feature set consists of mass radian, mean, maximum, median, standard deviation, skewness, kurtosis of gray level from spatial domain, energy, entropy, modified-entropy, contrast, inverse different moment, correlation, maximum, *SD_x_* (standard deviation) and *SD_y_* from the co-occurrence matrix of gray scale used *P_ϕ,d_*(*a*, *b*) with distance *d* =*1* and angle φ = 0°, 45°, 90°, 135° from texture domain and block activity, spectral entropy from the spectral domain. *Step* 1 of the experiment is to classify homogeneous features into 12 feature sets, using the bivariate correlation coefficient. Table 2 shows list of features in each set.

After Step 1, the simple and multiple logistic regression analysis in each feature set are performed. Tables 3 and 4 illustrate example results from Step 2 to Step 4 by using features in feature set #1.

Table 3 shows the effects among features in set #1. Values in Table 3 are used to test null hypotheses that two testing features are not correlated. If any effects that have *p*-value less than 0.05, those pairs of features are accepted as correlated.

Tables 3 and 4 show that:

- From Table 3: Entropy 0° and Entropy 45° are highly significantly related.
- From the second column of Table 4: based on the simple logistic model, only Entropy 0° causes **y** (Entropy 0° is significant to **y**).
- From the third column of Table 4: on the multiple logistic regression model, Entropy 0° and Entropy 45° cause **y**.
- Finally, with Bayes inference, the direct effect is Entropy 0° and the indirect effect is the interaction of Entropy 0° and Entropy 45° cause **y**.

**Figure 3.**
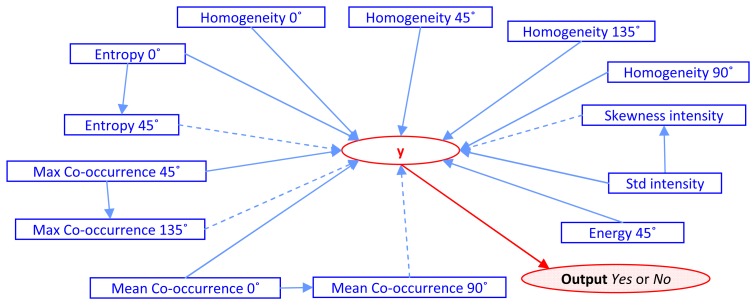
Complete graph on the experiment with direct and indirect effect from retaining process. (Dotted lines show indirect effects).

Table 4 shows the result of Step *4, D_i_* = *A_i_Ə B_i_* where *i* =1. After *k* iterations of the algorithm, the experiment results in the number of features being reduced from the original 50 to 1*3* features. Those features are Entropy 0°, Entropy 45°, Max Co-occurrence 45°, Max Co-occurrence 135°, Mean Co- occurrence 0°, Mean Co-occurrence 90°, Energy 45°, Homogeneity 0°, Homogeneity 45°, Homogeneity 90°, Homogeneity 135°, Standard deviation and Skewness of intensity value. The constructive cause and effect graph, *G*(*V*,*E*|**y**), is shown as Figure 3.

### 6.2. Verification

The effectiveness of our selected 13-feature set (our-13) is compared to the results of the all-feature set (all-50) and 26-feature set from SFS (SFS-26) on two learning systems: ANN and logistic regression. True positive (TP), false positive rate (FP) and minimum squared error (MSE) are metrics in the comparison. Tables 5 and 6 show the results from ANN and logistic regression, respectively. Both tables show that the effectiveness of our-13 is better than of SFS-26 and it is much closer to all- 50. This shows that our method can detect comparably the same results while the feature computation is reduced by half compared to SFS and 13/50 compared to using all features.

### 6.3. Analysis of results

Graph-based analysis was examined using statistical techniques to identify the crucial direct or indirect features for breast cancer detection in medical images. Our algorithm requires time complexity *O*(*n*^2^). We can accept the hypothesis that there is no significance between 50 features and 13 features for ANN and logistic regression with threshold 5%. A comparison of the performance between the different configurations of architectures over two set of features (50 and 13 features) with two classifiers (ANN and logistic regression) indicates that the selected 13 features provide the best results in terms of precision with respect to computation time. Using our approach, the detection step improves the temporal ratio of computation by number of features by 50:13. Moreover, the proposed method demonstrates satisfactory performance and cost compared to SFS.

In our experiment, the 50 features were partitioned into 12 feature sets with *S*_11_ being the largest set. With this set, the search space for direct cause features (*A*_7_) is (^7^*C*_1_) while indirect cause (*B*_7_) exploration was (^7^*Ci*) *i*=2, 3 … 7. We also found that there were 11 features from the texture domain and two features from the spatial domain that were eliminated from the selection process. The mass radian was not a significant feature because some masses on benign images were larger than on malignant images. Instead of using mass radian (microcalcification), the distribution of micro- calcification is more advantageous.

On the theoretical aspect of finding a best combination feature set, the only way to guarantee the selection of an optimal feature set is an exhaustive search of all possible subsets of features. However, the search space could be very large: 2*^N^* for a set of *N* features. Our algorithm provides a divide and conquer strategy; with *N* features (assume that there are *r* groups with *k* features each), the number of possible subsets for examining the feature selection is *r^k^C_i_*; *i*=1, 2 …*k*.

## 7. Conclusions

In this research, a method to reduce a number of features for medical image detection is proposed. We use mammograms from the Mammographic Image Analysis Society (MIAS) as test data and applied the proposed algorithm to reduce the number of features from a frequently-used 50 features to 13 features, while the accuracies using two learning models are substantially the same. Our method can reduce the computation cost of mammogram image analysis and can be applied to other image analysis applications. The algorithm uses simple statistical techniques (path analysis, simple logistic regression, multiple logistic regressions, and hypothesis testing) in collaboration to develop a novel feature selection technique for medical image analysis. The value of this technique is that it not only tackles the measurement problem by path analysis but also provides a visualization of the relation among features. In addition to ease of use, this approach effectively addresses the feature redundancy problem. The method proposed has been proven that it is easier and it requires less computing time than using SFS, SBF and genetic algorithms. For further research, a deeper analysis of the texture domain and the dispersion of microcalcification may provide a more efficient breast CAD system, with cost reduction and higher precision.

## Figures and Tables

**Table 1.** Feature selection and classification method from previous work.

Researcher	Domain	Features used (examples)	Classifier
Fu *et al.* [13]	Texture	Co-occurrence matrix rotation with angle 0°, 45°, 90°, 135°: Difference entropy, entropy, difference variance, contrast, angular second moment, correlation	GRNN (SFS, SBS)
	Spatial	Mean, area, standard deviation, foreground/ background ratio, area, shape moment intensity variance, energy –variance	
	Spectral	Block activity, Spectral entropy	

G. Samuel *et al.* [5]	Spatial	Volume, sphericity, mean gray level, gray level standard deviation, gray level threshold, radius of sphere, maximum eccentricity, maximum circularity, maximum compactness	Rule-based, linear discriminant analysis

E. Lori *et al.* [4]	Spatial, Patient Profile	Patient profile, nodule size, shape (measured with ordinal scale)	Regression analysis

Shiraishi *et al.* [12]	Multi Domain	Patient profile, root-mean-square of power spectrum,histograms frequency, full width at half maximum of the histogram for the outside region of the segmented nodule on the background–corrected image, degree of irregularity, full width at half maximum for inside region of segmented nodule on the original image	Linear discriminant analysis

Hening [18]	Spatial	Average gray level, standard deviation, skew, kurtosis, min- max of the gray Level, gray level histogram	SVM

Zhao *et al.* [27]	Spatial	Number of pixels, histogram, average gray, boundary gray, contrast, difference, energy, modified energy, entropy, standard deviation, modified standard deviation, skewness, modified skewness	ANN

Ping *et al.* [21]	Spatial	Number of pixels, average, average gray level, average histogram, energy, modified energy, entropy, modified entropy, standard deviation, modified standard deviation, skew, modified skew, difference, contrast, average boundary gray level	ANN and Statistical classifier

Songyang and Ling, [24]	Mixed features	Mean, standard deviation, edge, background, foreground- background ratio, foreground-background difference, difference ratio of intensity, compactness, elongation, Shape Moment I-IV, Invariant Moment I-IV, Contrast, area, shape, entropy, angular second moment, inverse different moment, Correlation, Variance, Sum average	Multi-layer Neural Network

**Table 2.** Partition of the 50 original features into 12 feature sets.

**Feature set**	**Number of features**	**List of Features**
#1	4	Entropy rotations from 0°, 45°, 90°, 135°
#2	4	Energy rotations from 0°, 45°, 90°, 135°
#3	4	Inverse difference Moment rotations from 0°, 45°, 90°, 135°
#4	4	Mean Co-occurrence rotations from 0°, 45°, 90°, 135
#5	4	Max Co-occurrence rotations from 0°, 45°, 90°, 135
#6	4	Contrast rotations from 0°, 45°, 90°, 135°
#7	4	Homogeneity rotations from 0°, 45°, 90°, 135°
#8	4	Standard deviations on X rotation from 0°, 45°, 90°, 135°
#9	4	Standard deviations on Y rotation from 0°, 45°, 90°, 135°
#10	4	Modified entropy rotations from 0°, 45°, 90°, 135°
#11	7	mean, maximum, median, standard deviation (SD), coefficient of variation (CV), skewness, kurtosis (intensity of gray level)
#12	3	block activity, spectral entropy, mass radian

**Table 3.** The effects among features in feature set #1.

**Relations in Feature set #1**	**Effects of dependent features (using simple linear regression)**
Entropy 0° to Entropy 45°	0.000 **
Entropy 0° to Entropy 90°	0.004 *
Entropy 0° to Entropy 135°	0.000 *
Entropy 45° to Entropy 90°	0.000 **
Entropy 45° to Entropy 135°	0.022 *
Entropy 90° to Entropy 135°	0.000 **

* denotes significant with 5% threshold and

** denotes highly significant with 1% threshold.

**Table 4.** The effects of features in feature set #1 on output.

**Feature set #1**	**Effects on output**	

	**Using simple logistic regression**	**Using multiple logistic regression**
Entropy 0°	0.034 *	0.026 *
Entropy 45°	0.433	0.031 *
Entropy 90°	0.363	0.241
Entropy 135°	0.159	0.169

* denotes significant with 5% threshold and

** denotes highly significant with 1% threshold.

**Table 5.** Performance of logistic regression using all-50, SFS-26 and our-13 feature sets.

**Logistic regression**	**TP (%)**	**FP (%)**	**MSE**
Using original 50 features (all-50)	82.94	14.51	0.052
Using selected 26 features (SFS-26)	77.41	18.72	0.102
Using selected 13 features (our-13)	81.64	15.06	0.084

**Table 6.** Performance of ANN using all-50, SFS-26, and our-13 feature sets.

**ANN**	**TP (%)**	**FP (%)**	**MSE**
Using original 50 features (all-50)	83.32	14.42	0.034
Using selected 26 features (SFS-26)	78.59	16.02	0.083
Using selected 13 features (our-13)	82.35	15.02	0.065
